# Unraveling Reality : Complaints that became delusions aligned to Ehlers-Danlos Syndrome ended up with ECT - a case report

**DOI:** 10.1192/j.eurpsy.2025.2134

**Published:** 2025-08-26

**Authors:** D. Uslu, R. E. Dindar, E. E. Bestepe, H. Kolsuz

**Affiliations:** 1 Psychiatry, Istanbul Erenköy Mental and Nervous Diseases Training and Research Hospital, Istanbul, Türkiye

## Abstract

**Introduction:**

Ehlers-Danlos Syndrome (EDS) is a group of hereditary connective tissue disorders that primarily involve skin hyperelasticity, hypermobility of joints and fragility of blood vessels.This syndrome shows heterogeneous features.Recent studies have shown that patients with EDS have a higher risk of psychiatric conditions such as depression, suicide and schizophrenia.The existing literature on safe administration of ECT defines several case reports which incorporates connective tissue disorders.

**Objectives:**

This case report aims to define a rare case of EDS related psychosis including the clinical presentation and management.Furthermore, to show the administration of ECT in patients suffer from this medical condition after their assessment.

**Methods:**

Comprehensive presentation of patient’s case and review of systematic literature using database, in regards to connective tissue disorders and psychiatric conditions.

**Results:**

A 42-year-old female patient,diagnosed with anxiety disorder 7 years prior, who has shown no remission and has experienced multiple hospital admissions over the past year due to various psychotic and depressive episodes, presented to our hospital with complaints that a “sorcerer” had cast spells on her.She reported delusions of mystical and somatic nature, describing that as a result of these spells, supernatural entities had caused her teeth to shrink, her skull to shift, the brightness in her eyes to diminish, her internal organs to decay and her physical presence to fade away.Additionally, she expressed paranoid-persecutory delusions that these entities would ultimately lead to her death.Treatment history included multiple SSRIs, antipsychotics and 6 sessions of TMS therapy, all with limited response.Her family history revealed that her father had died by suicide and her siblings were also diagnosed with depression/anxiety disorders.

The patient was admitted with a preliminary diagnosis of psychotic depression.Physical examination findings included hyperflexibility and hyperlaxity, suggestive of EDS.As the patient was at increased theoretical risk for complications; several consultations were requested to assess fitness for treatment prior to ECT.Based on these evaluations, ECT was deemed safe to proceed.Bifrontal ECT was successfully administered, resulting in marked improvement in the patient’s psychotic symptoms.

**Conclusions:**

Even delusions are remarkably indicative for psychosis, it is crucial to recognize the underlying medical conditions may contribute to its onset and/or exacerbation.Psychiatric evaluations should be performed during EDS patients’ routine follow-ups.Additionally, as is already shown in other case reports, administration of ECT in patients with EDS should be considered.However, further research is required to understand the eligibility of ECT on connective tissue disorders also to expand our repertoire in the treatment.

**Disclosure of Interest:**

None Declared

